# Minimally invasive approaches using virtual reality planning in elective aneurysm surgery

**DOI:** 10.3389/fsurg.2025.1713243

**Published:** 2025-12-04

**Authors:** Ladina Greuter, Tabea Stoessel, Florian Ebel, Emilia Westarp, Attill Saemann, Jonathan Rychen, Philippe Cattin, Marek Zelechowski, Balasz Faludi, Jehuda Soleman, Raphael Guzman

**Affiliations:** 1 Department of Neurosurgery, University Hospital Basel, Basel, Switzerland; 2 Center for Medical Image Analysis & Navigation (CIAN), University of Basel, Basel, Switzerland; 3 Faculty of Medicine, University of Basel, Basel, Switzerland

**Keywords:** virtual reality, aneurysm surgery, neurovascular surgery, minimally invasive approach, SpectoMedical®

## Abstract

**Introduction:**

While endovascular treatment has gained popularity for its minimally invasive approach, microsurgical clipping for unruptured intracranial aneurysms (UIAs) has also evolved using surgical adjuncts such as virtual reality (VR) and keyhole techniques to enhance patient outcomes. Immersive 3D VR images allow for the creation of an accurate 3D anatomical model, making it a valuable tool for surgical planning. This study investigated the impact of VR-based surgical planning on approach type and craniotomy size.

**Methods:**

This retrospective cohort study included all patients who underwent elective microsurgical clipping for UIAs from 1 January 2009 to 31 December 2024 at the University Hospital of Basel, Switzerland, and was approved by the local ethics board. Demographic, surgical, and outcome parameters were collected. SpectoMedical®, developed at the University of Basel, was the VR platform used. The primary outcome was craniotomy size (cm^2^) measured using the anterior-posterior method. Descriptive and comparative statistics were conducted. To assess the factors influencing the craniotomy size, we calculated a multivariable linear regression model.

**Results:**

We included a total of 163 aneurysms in 159 patients with a mean age of 58.52 (±10.23), and 114 (69.9%) were female. VR-based surgical planning resulted in a significantly smaller craniotomy size [no VR vs. VR, 20.31 (±19.21) cm^2^ vs. 13.22 (±7.85) cm^2^, *p* = 0.007] and shorter hospital stay [no VR vs. VR, 10.04 (±5.58) vs. 7.89 (±2.79) days, *p* = 0.031]. Operative time was shorter in the VR group but lacked statistical significance [no VR vs. VR, 226.41 (±86.18) min vs. 207.93 (±54.92) min, *p* = 0.160]. The multivariable regression model showed that the use of VR-based surgical planning reduced the craniotomy size by 6.2 cm^2^.

**Conclusion:**

VR-based surgical planning was associated with significantly smaller craniotomy sizes and shorter hospital stays. It results in an intraoperative déjà-vu effect for the surgeon, which supports its use as a valuable adjunct in preoperative planning.

## Introduction

Unruptured intracranial aneurysms (UIAs) have a prevalence of approximately 2.8%, with most of them found incidentally (1, 2). Treatment decisions are guided by individualized risk stratification, considering patient- and aneurysm-specific characteristics. Patients undergoing surgery for UIAs are typically neurologically intact, leaving a narrow margin for complications. With endovascular treatment (EVT) gaining popularity due to its minimally invasive nature, microsurgical clipping for UIAs has become increasingly rare over the last decade, despite its superior long-term occlusion rate (3).

Over the last decade, microsurgical clipping has evolved through minimally invasive approaches and technological adjuncts, including intraoperative imaging, indocyanine green angiography, and virtual or augmented reality tools, aiming to reduce morbidity and shorten recovery time (4–12).

Virtual reality (VR) platforms have become a valid adjunct for surgical planning and training, with improved learning curves and shorter operating times for microsurgical clipping (13–16). Traditionally, aneurysm surgery was planned based on conventional two-dimensional (2D) images, requiring the surgeons to rely on their three-dimensional (3D) vascular anatomy abstraction; however, immersive 3D VR images can facilitate this process, especially for novice learners, creating a valuable tool for surgical planning, skills training, and education (13). However, there are only a few studies available comparing outcomes using VR as a standard tool for aneurysm surgery (14, 16, 17).

This study aimed to compare VR-based surgical planning to conventional surgical planning using 2D images (control group) regarding minimally invasive approaches and craniotomy size.

## Methods

We included all patients who underwent elective microsurgical clipping for UIAs from 1 January 2009 to 31 December 2024 at the University Hospital of Basel, Switzerland. The study complied with the Helsinki Guidelines and was approved by the local ethics board (EKNZ Basel, Switzerland, 2021-01390 and 2018-01705). Patients with previously ruptured and secured aneurysms requiring elective retreatment were also included in this study. If the same patient had multiple clippings for different aneurysms, the patient was included multiple times. Patients presenting with subarachnoid hemorrhage (SAH) due to ruptured aneurysms were excluded, given that their management and outcomes differ vastly from elective aneurysm treatment.

All clinical decisions were discussed in our weekly multidisciplinary vascular board, which includes fellowship-trained neurovascular surgeons and neurointerventionalists. All the surgeries were performed or supervised by a fellowship-trained neurovascular surgeon. Patients typically underwent preoperative digital subtraction angiography (DSA). If this was not possible, alternative imaging, including computer tomography angiography (CTA) or magnetic resonance imaging (MRI), was obtained.

We collected demographic data (age, sex), the location and maximal diameter (mm) of each aneurysm, and how many aneurysms a patient harbored. We further recorded if the patient had a previous subarachnoid hemorrhage or if the aneurysm the patient underwent surgery for had already been treated, and if so, with which modality (EVT vs. microsurgical clipping).

The primary endpoint for surgical outcomes was the craniotomy size (cm^2^) due to its link to recovery time and cosmetic outcomes (12, 17). The surface area of the craniotomy was calculated using the anterior–posterior (AC) method, in which the area is calculated by multiplying the craniectomy length (*A*) by its height (*C*) and which has been previously described in detail by Ho et al. (18). As a standard of care in our institution, the patients received a postoperative CTA after aneurysm clipping, which was used to calculate the craniotomy surface area on an axial bone window. Additional surgical details included the approach used (pterional, minipterional, keyhole supraorbital, keyhole sylvian fissure, subtemporal, or suboccipital), operating time (min), and how many aneurysms were clipped in one surgery.

From 2018 onwards, regular intraoperative DSA was conducted to confirm complete aneurysm occlusion, if available, and was automatically added to the operative (OR) time. Thus, for patients who underwent an intraoperative DSA, we deducted 45 min from their overall operative time.

As clinical outcome parameters, we collected pre- and postoperative Glasgow Coma Scale (GCS) scores and perioperative complications, which were defined as complications occurring either intraoperatively or during hospitalization. Data on complications were collected in general, but we further distinguished between hemorrhagic complications requiring either a repeat surgery or causing a new neurological deficit, ischemic complications leading to a new neurological deficit, and others (e.g., seizures, wound infection, pseudomeningocele, cranial nerve palsy, and unsatisfactory aneurysm occlusion). Temporary deficits were defined as a new postoperative neurological deficit, from which the patient completely recovered within the first year after surgery.

Patients had a postoperative follow-up at approximately 6 weeks and a final follow-up at 1 year after microsurgical clipping in the case of a singular aneurysm. If the patients harbored more aneurysms, annual follow-ups were continued.

### Surgical planning using VR

From 2018 onwards, we used VR as a standard for elective aneurysm surgery planning, regardless of any individual patient parameters or the complexity of the case.

We used SpectoMedical® (Specto Medical AG, Basel, Switzerland) as the VR platform to plan the aneurysm surgeries. SpectoMedical® was developed at the Department of Biomedical Engineering, University of Basel, Switzerland, and carries a *Conformité Européene* (CE) mark as a Class 1 medical device (SpectoMedical® AG, Basel, Switzerland). It renders a patient-specific, fully immersive 3D VR model based on Digital Imaging and Communications in Medicine (DICOM) data. Either preoperative CTA or DSA (Dyna CTA) images were used to render the VR model. No additional images were used for the image rendering and only images that were clinically indicated were used. The platform’s anatomical and geometrical accuracy has been verified in previous medical studies (19–21). A fully immersive VR experience was created using a commercial VR headset system (HCT Vive® headset and controllers, Valve Corporation, Bellevue, Washington, DC, USA).

**Figure 1:**
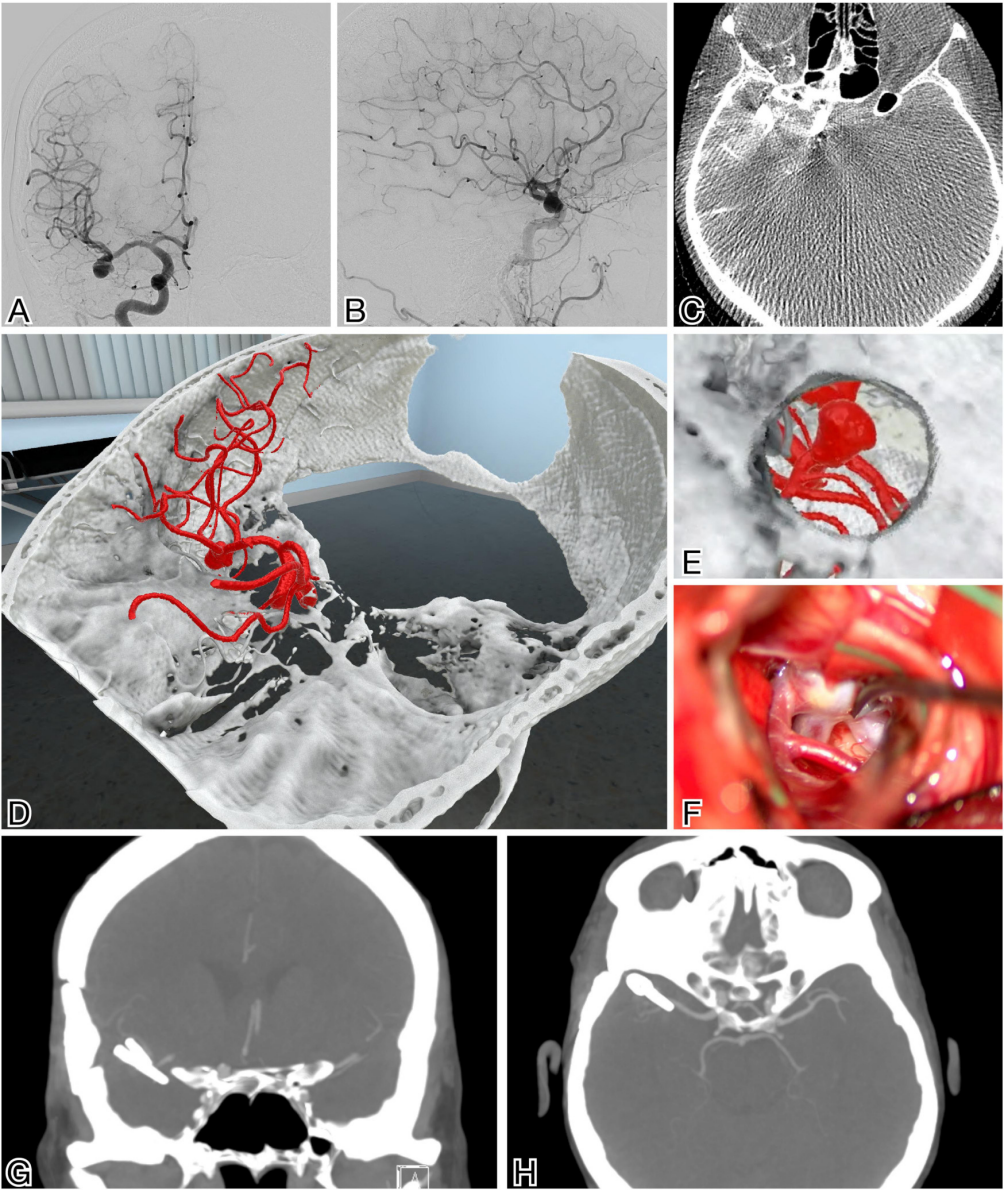
MCA aneurysm planning using VR as an adjunct for surgical planning. **(A,B)** Frontal and lateral views of the MCA aneurysm in DSA. **(C)** Dyna-CT with an arterial injection of the right internal carotid artery is used as input data for the VR reconstruction **(D)**. Visualization of the aneurysm through the planned customized approach **(E)** in Specto Medical®. **(F)** Intraoperative view of the aneurysm with some of its anatomy covered and preoperatively analyzed in VR. **(G,H)** Postoperative CTA showing complete occlusion of the aneurysm.

Preoperatively, the surgical team prepared for the microsurgical clipping procedure by studying and refining the positioning of the patient; the surgical strategy, including branching vessels and small perforators; and the angle of clip placement based on the VR model (Figure 1, Supplementary Video S1). Studying the VR model in detail allowed the surgical team to rehearse the procedure in a fully immersive virtual operating room.

The surgical approaches were mainly selected based on the aneurysm’s location, size, morphology, and anatomical orientation, as well as patient-specific factors, and those selected are standard approaches for aneurysm clipping (11, 22–24).

### Statistical analysis

We conducted descriptive statistics, calculating the mean ± standard deviation for continuous variables and median (interquartile range) for ordinal data.

Posterior circulation aneurysms were excluded from the comparative statistics to avoid any bias, given the low number of included aneurysms [*n* = 3, one in the posterior inferior cerebellar artery (PICA) and two in the posterior cerebral artery (PCA)]. We further combined the different anterior cerebral artery (ACA) (e.g., A2 or A3) and internal carotid artery (ICA) aneurysms (e.g., supraophthalmic, paraophthalmic, ICA terminus, or anterior choroidal) into one group.

We further dichotomized the patient cohort according to the use of VR for surgical planning, craniotomy and aneurysm location. A multivariable linear regression model based on clinically relevant parameters and significant variables from the univariate analysis was calculated for the primary outcome.

All the statistical analyses were conducted in R (R software for statistics, 2024.12.0 + 467, Posit software), and a *p*-value of <0.05 was considered statistically significant.

## Results

### Demographics

We included a total of 163 aneurysms in 159 patients, with a mean age of 58.52 (±10.23) and 114 (69.9%) female patients and 49 (30.1%) male patients. The mean preoperative GCS score in our cohort was 14.96 (±0.26). Middle cerebral artery (MCA) aneurysms were the most frequently treated, comprising 57.7% (*n* = 94) of all the cases, followed by anterior communicating artery (Acom) aneurysms, which comprised 25.2% (*n* = 41) of all cases (Table 1). The mean aneurysm diameter was 7.41 (±3.24) mm (Table 1). In total, 16 patients (9.8%) suffered from a previous subarachnoid hemorrhage and required retreatment for either the same or another aneurysm. Moreover, 12 patients (7.5%) had already undergone previous treatment for the same aneurysm, of which nine (5.6%) had undergone a previous EVT and three (1.9%) a previous microsurgical clipping. The mean time to retreatment from the previous aneurysm for both EVT and microsurgical clipping was 5.50 (±4.68) years.

**Table 1:** Demographic information.

Parameter	Number
*n*	163
Sex = male (%)	49 (30.1)
Sex = female (%)	114 (69.9)
Age [mean (±SD)] (years)	58.52 (10.23)
Previous hemorrhage = yes (%)	16 (9.8)
VR = yes (%)	66 (40.5)
Aneurysm (%)	
A2	1 (0.6)
A3	3 (1.8)
AchoA	2 (1.2)
Acom	41 (25.2)
ICA paraophtalmic	3 (1.8)
ICA supraophthalmic	1 (0.6)
ICA terminus	5 (3.1)
MCA	94 (57.7)
PCA	2 (1.2)
Pcom	10 (6.1)
PICA	1 (0.6)
Aneurysm size [mean (±SD)] (mm)	7.41 (3.24)
GCS preoperative [mean (±SD)]	14.96 (0.26)

VR, virtual reality; AchoA, anterior choroidal artery; Acom, anterior communicating artery; ICA, internal carotid artery; MCA, middle cerebral artery; PCA, posterior cerebral artery; Pcom, posterior communicating artery; PICA, posterior inferior cerebellar artery; SD, standard deviation.

### VR-based surgical planning for microsurgical clipping compared to conventional surgical planning

**Figure 2:**
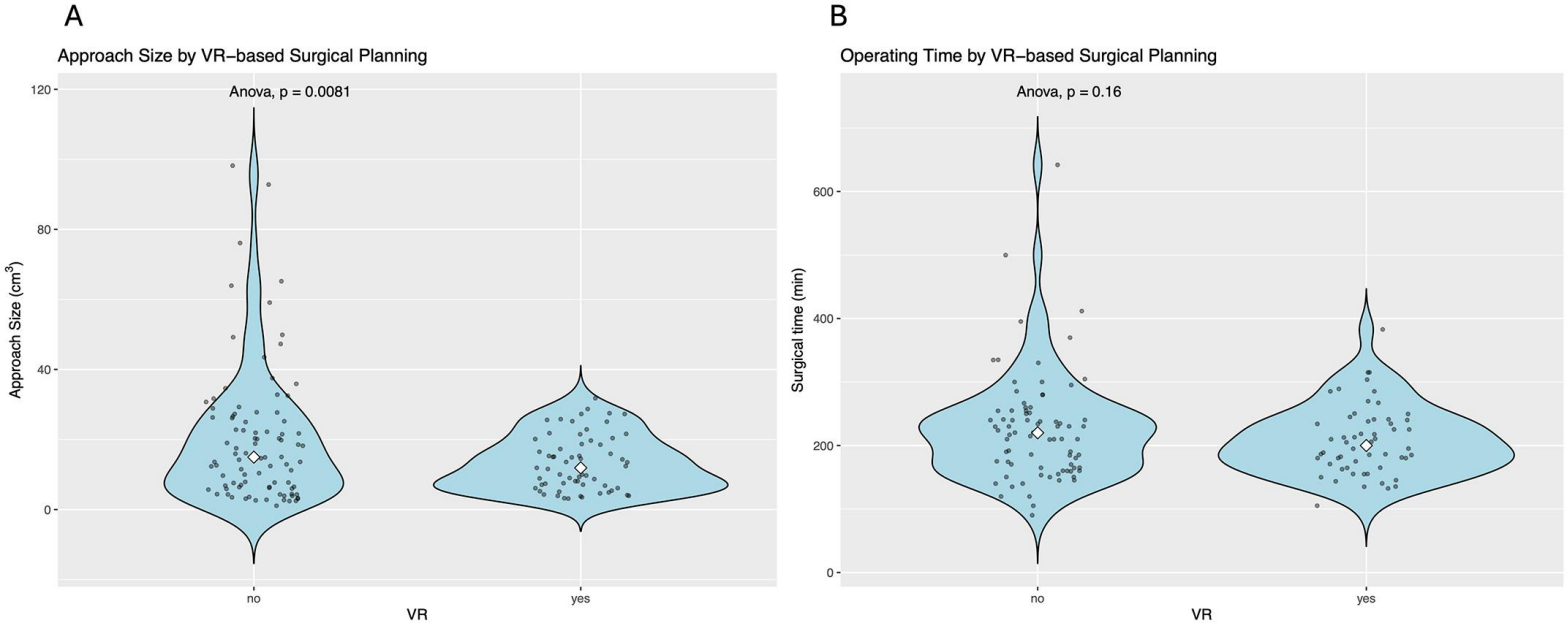
**(A)** Approach size is significantly smaller in patients with VR-based surgical planning, while **(B)** operating time is not associated with VR-based surgical planning.

VR was used for surgical planning in 40.5% (*n* = 64) compared to conventional surgical planning in 59.5% (*n* = 97). No difference in the baseline demographic data, such as age, sex, aneurysm location, or aneurysm size (Table 2), was detected between the two groups. The type and size of craniotomy differed significantly between the two groups (Table 2). Minipterional and sylvian keyhole craniotomies were used significantly more in the VR group [minipterional no VR vs. VR, 10 (10.4%) vs. 41 (64.1%), *p* < 0.001; sylvian keyhole no VR vs. VR, 0 (0.0%) vs. 9 (14.1%), *p* < 0.001, Table 2]. Supraorbital approaches were performed significantly less often in the VR group [no VR vs. VR, 14 (14.6%) vs. 2 (3.1%), *p* < 0.001, Table 2]. Furthermore, using VR-based surgical planning led to a significantly smaller craniotomy size [no VR vs. VR, 20.31 (±19.21) cm^2^ vs. 13.22 (±7.85) cm^2^, *p* = 0.007, Table 3, Figure 2A]. Surgical duration was shorter in the VR group but lacked statistical significance [no VR vs. VR, 226.41 (±86.18) min vs. 207.93 (±54.92) min, *p* = 0.160, Table 2, Figure 2B].

**Table 2:** Demographics and outcome dichotomized by the use of VR for surgical planning.

Parameter	VR no	VR yes	*p*-Value
*n*	96	64	
Age [mean (±SD)] (years)	57.87 (10.27)	59.84 (9.86)	0.23
Sex = male (%)	31 (32.3)	18 (28.1)	0.7
GCS preoperative score [mean (±SD)]	14.95 (0.30)	14.98 (0.16)	0.581
Previous hemorrhage = yes (%)	11 (11.5)	5 (7.8)	0.628
Aneurysm (%)			0.17
ACA	2 (2.1)	2 (3.1)	
Acom	27 (28.1)	14 (21.9)	
ICA	10 (10.4)	1 (1.6)	
MCA	51 (53.1)	43 (67.2)	
PCOM	6 (6.2)	4 (6.2)	
Aneurysm [mean (±SD)] (mm)	7.46 (3.46)	7.11 (1.96)	0.467
Craniotomy approach (%)			**<0** **.** **001**
Frontal	2 (2.1)	2 (3.1)	
Keyhole sylvian	0 (0.0)	9 (14.1)	
Minipterional	10 (10.4)	41 (64.1)	
Pterional	70 (72.9)	10 (15.6)	
Supraorbital	14 (14.6)	2 (3.1)	
Approach size [mean (±SD)] (cm^2^)	20.31 (19.21)	13.22 (7.85)	**0** **.** **007**
Operating time [mean (±SD)] (min)	226.41 (86.18)	207.93 (54.92)	0.16
GCS postoperative score [mean (±SD)]	14.89 (0.66)	14.97 (0.18)	0.334
Overall complications = yes (%)	7 (7.3)	9 (14.1)	0.259
Hemorrhagic complications = yes (%)	3 (3.1)	1 (1.6)	0.918
Ischemic complications = yes (%)	2 (2.1)	2 (3.1)	1
Other complications = yes (%)	2 (2.1)	6 (9.4)	0.089
Permanent neurological deficit = yes (%)	5 (5.2)	1 (1.6)	0.445
Hospitalization time [mean (±SD)] (days)	10.04 (5.58)	7.89 (2.79)	**0** **.** **031**

Bold values indicate statistically significant values.

**Table 3:** Outcome parameters.

Parameter	Number
*n*	163
Craniotomy approach (%)	
Frontal	4 (2.5)
Keyhole	9 (5.5)
Minipterional	51 (31.3)
Pterional	80 (49.1)
Suboccipital	1 (0.6)
Subtemporal	2 (1.2)
Supraorbital	16 (9.8)
Craniotomy size [mean (SD)] (cm^2^)	18.40 (19.92)
Number of aneurysms clipped during surgery [mean (±SD)]	1.07 (0.26)
Surgical time [mean (±SD)] (min)	218.74 (75.13)
GCS postoperative score [mean (±SD)]	14.91 (0.53)
Complications = yes (%)	16 (10)
Hemorrhagic complication = yes (%)	4 (2.5)
Ischemic complication = yes (%)	4 (2.5)
Other complications = yes (%)	8 (5.0)
Permanent neurological deficit = yes (%)	6 (3.8)
Hospitalization duration [mean (±SD)] (days)	9.56 (5.05)

Postoperative clinical status, as measured by the GCS, was comparable between the two groups [no VR vs. VR, 14.89 (±0.66) vs. 14.97 (±0.18), *p* = 0.334]. One patient (1.6%) in the VR group developed permanent neurological deficits compared to five patients (5.2%) in the conventional planning group [no VR vs. VR, 5 (5.2%) vs. 1 (1.6%), *p* = 0.445, Table 2]. The hospitalization duration was significantly shorter in the VR group [no VR vs. VR, 10.04 (±5.58) vs. 7.89 (±2.79) days, *p* = 0.031, Table 2].

### Multivariable linear regression model assessing the factors influencing craniotomy size

**Figure 3:**
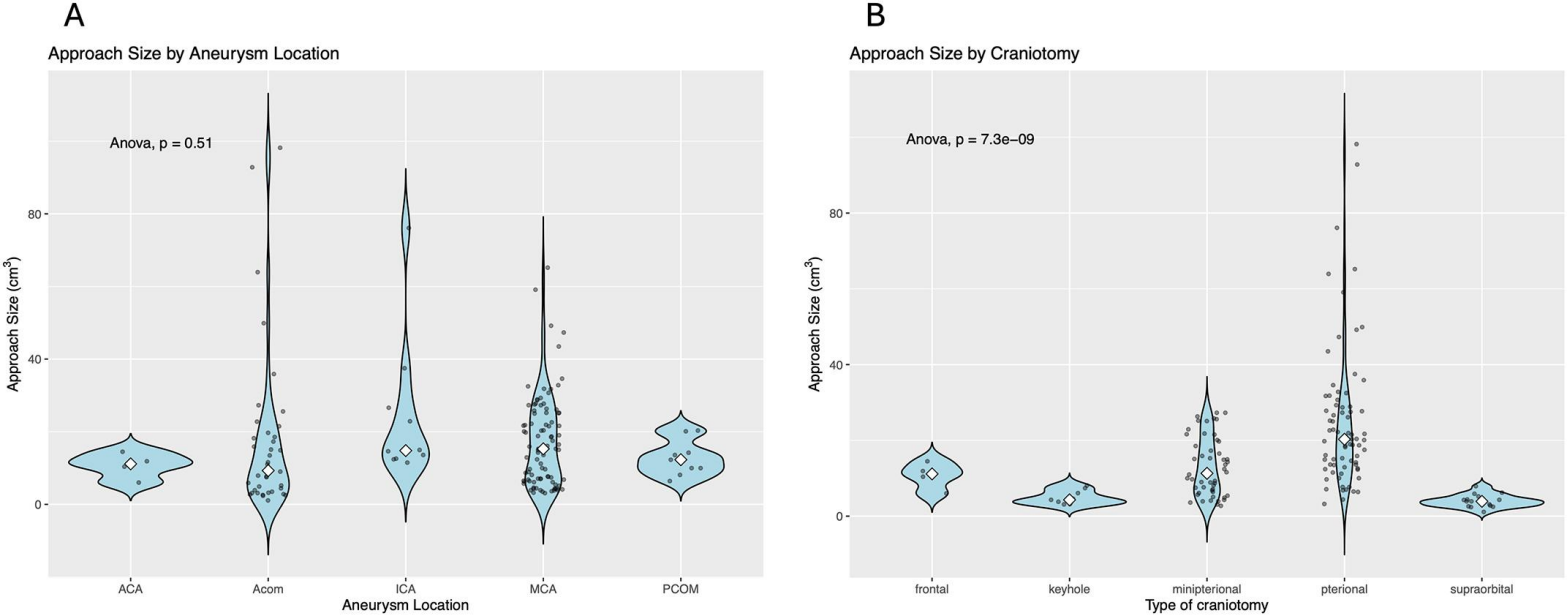
**(A)** Aneurysm location did not significantly influence the craniotomy size. **(B)** The type of craniotomy is significantly associated with craniotomy size (*p* < 0.001).

Aneurysm location did not significantly influence the craniotomy size (Figure 3A); however, the type of craniotomy was significantly associated with craniotomy size (Figure 3B, *p* < 0.001).

Furthermore, the use of VR-based surgical planning was significantly associated with a smaller craniotomy size (*β* = –6.20, *p* = 0.019), independent of patient age, aneurysm location, and bleeding history. Age was also an independent predictor, with increasing age associated with a modest reduction in approach size (*β* = –0.32, *p* = 0.017). No significant associations were found for aneurysm location or prior subarachnoid hemorrhage (Table 4). Finally, the regression model showed that the use of VR-based surgical planning reduced the craniotomy size by 6.2 cm^2^.

**Table 4:** Multivariable linear regression model assessing the association with craniotomy size.

Predictor	Estimate (*β*)	Std. error	t-Value	95% confidence interval	*p*-Value
(Intercept)	30.5888	10.6318	2.877	(9.58, 51.60)	0.00462
Acom aneurysm	7.9670	8.2211	0.969	(−8.27, 24.20)	0.33411
ICA aneurysm	9.8147	9.3439	1.050	(−8.64, 28.27)	0.29529
MCA aneurysm	8.3099	7.9916	1.040	(−7.48, 24.10)	0.30015
Pcom aneurysm	4.0584	9.4288	0.430	(−14.55, 22.67)	0.66752
VR (yes)	−6.2024	2.6234	−2.364	(−11.39, −1.01)	**0** **.** **01939**
Previous bleed (yes)	1.8898	4.4249	0.427	(−6.85, 10.63)	0.66996
Age (per year)	−0.3163	0.1305	−2.424	(−0.57, −0.06)	**0** **.** **01659**

Bold values indicate statistically significant values.

## Discussion

To our knowledge, this is the largest cohort study investigating VR-based surgical planning for microsurgical clipping, including a total of 163 UIAs (14, 16, 17, 25). VR-based aneurysm surgery planning resulted in a significantly smaller craniotomy size and shorter hospital stay (Table 2).

Operating time was shorter in the VR group but lacked statistical significance (Table 2, Figure 2). In the multivariable regression model, the use of VR and age were significantly associated with a smaller craniotomy size, while the aneurysm location or whether a previous SAH was present failed to show an association (Table 4). The majority of the patients included in this study underwent surgery for MCA aneurysms (57.7%, *n* = 94), with Acom aneurysms being the second most common (25.2%, *n* = 41).

In recent decades, endovascular treatment for aneurysms has become more widespread, as it is a less invasive technique and is usually associated with shorter hospital stays compared to open microsurgical clipping (3, 8). However, many aneurysms in the anterior circulation still require microsurgical clipping, with superior long-term outcomes (3). Microsurgical clipping has also drastically evolved due to technical advances, with adjuncts such as micro-Doppler imaging, flow probes, indocyanine green video angiography, and augmented and virtual reality tools aiming to improve outcomes and the procedure being more minimally invasive (4, 5, 8, 10, 22).

The gold standard approach to an aneurysm in the anterior circulation is pterional craniotomy. Since its first modern description by Yaşargil in the 1970s, pterional craniotomy has been the workhorse for aneurysm clipping for UIAs and ruptured aneurysms (23). It allows for an excellent visualization of the anterior and posterior circulations, the sphenoid wing, the cavernous sinus, and the sellar/parasellar region, serving as the gold standard approach to these regions (12, 22). However, over time, drawbacks such as temporal muscle atrophy, requiring a long incision, or facial nerve injury have arisen, leading to modified approaches (12, 22, 26–28). As an evolution, minipterional craniotomy was first described by Figueiredo et al. (22). It allows for an optimal corridor to the sylvian fissure but centers around the pterion, omitting the often-unnecessary exposure of the distal sylvian fissure (22). It leads to less soft tissue trauma and requires a significantly smaller bony opening, which was confirmed in our series [minipterional: 13.07 (±7.69) cm^2^ vs. pterional: 25.18 (±18.85) mm^2^, *p* < 0.001] (22). Less soft tissue trauma could possibly improve mastication and hence positively impact patient outcomes. A systematic review comparing minipterional to pterional craniotomy showed that minipterional craniotomy leads to improved cosmetic and functional outcomes, lowers morbidity, and a shorter operating time (12). Hence, minipterional craniotomy has increasingly gained popularity for accessing anterior circulation aneurysms, reserving pterional craniotomy for ruptured cases, where a larger opening to place an external ventricular drain or evacuate a hematoma may be required (29). Our series confirmed that, over time, a shift from the standard pterional to the minipterional craniotomy occurred, with significantly smaller approaches when VR was used for surgical planning. VR can help plan minimally invasive approaches, facilitating this shift from conventional pterional to minipterional craniotomy. In addition to the development of minipterional craniotomy, other minimally invasive approaches have been developed over time, such as the supraorbital or lateral supraorbital approach (11, 29–31). The lateral supraorbital approach (LSO) was first described by Hernesniemi et al. and provides subfrontal access to the sylvian fissure compared to the minipterional craniotomy, which has a more lateral trajectory (30, 32). Both have been shown to provide an excellent access corridor and outcomes for anterior circulation aneurysm clipping (4, 30, 32–34). A prospective randomized trial comparing pterional craniotomy to keyhole approaches confirmed their superiority regarding cosmetic and quality-of-life outcomes and operating time (31). Keyhole approaches with VR-based surgical planning resulted in a 20-min shorter operating time; however, this difference lacked statistical significance. However, VR-based surgical planning allowed us to achieve a significantly smaller craniotomy size and shorter hospital stay. In other studies, shorter operating time has been shown to correlate with lower infection and complication rates, which could have direct clinical benefit for patients (35, 36). Furthermore, VR-based operative planning allows the surgeon to confidently plan a keyhole approach, as the spatial anatomy, especially the branching vessels and dome orientation, and possible adhesions can be studied beforehand (17). VR-based surgical planning has been confirmed to improve microsurgical clipping outcomes in multiple studies (13–16). In our experience, when using VR to meticulously plan the surgical set-up, including the dissection of branching vessels and clip placement angle, an intraoperative déjà-vu effect for the surgeon is achieved. This facilitates the dissection of relevant structures, even if they may initially be hidden in the real-life surgical field, and can increase the surgeon's orientation and confidence (16, 37). However, measuring the intraoperative déjà-vu effect directly is cumbersome, which is why indirect outcome measures, such as operating time, clip placement time, or approach size, have been chosen (14, 16, 17).

A randomized study by Chugh et al. comparing surgical time and number of clip placement attempts between surgeons who prepared for surgery with preoperative VR planning compared to conventional methods failed to show a difference in the overall duration of surgery; however, they found that a significantly shorter time was required for clip placement in the VR group (14). A similar study by Steineke et al. showed a reduction in the overall surgical time when using VR-based surgical planning, confirming it to be a more efficient and safe surgical approach (16). This was confirmed by another study that described a VR-guided keyhole sylvian approach, which was found to be superior to the conventional pterional approach (17). Similar to the studies in the literature, we showed a reduction in the operating time when using VR; however, this lacked statistical significance. Moreover, we did not assess the time required for clip placement, given the retrospective nature of this study. An important difference to note is that other studies only included MCA aneurysms, while we included all aneurysms in the anterior circulation. We showed that the aneurysm's location does influence the surgical time (Supplementary Figure S2). We further demonstrated that VR-based surgical planning resulted in a significantly smaller craniotomy size and shorter hospital stay, while other studies did not analyze the craniotomy size or failed to show a difference in hospitalization time (14, 16). Craniotomy size remains an important outcome, given that a smaller size is related to less soft tissue manipulation, and keyhole approaches have been shown to improve patient outcomes and satisfaction (8, 10, 17, 29). The significantly shorter hospital stay in the VR group could be explained by improved perioperative management in this group, as the control group was treated 5–10 years prior, and may not be directly related to the use of VR (38).

Apart from VR-based surgical planning, age was shown to be significantly associated with a smaller craniotomy size. This could be explained by brain atrophy and larger subarachnoid spaces, allowing for more cerebrospinal fluid release than in younger patients, resulting in a smaller bony opening being required to achieve comparable surgical trajectories (39, 40). Another factor explaining this association could be that microsurgical clipping has become safer over the years and, due to the aging of the population, microsurgical clipping is offered more readily to elderly patients (39).

Despite their smaller size, keyhole approaches with VR-based surgical planning are safe, with a low permanent deficit rate of 1.6% (12, 29).

To further investigate the advantages of VR-based surgical planning for clip placement, we are currently conducting a prospective trial at our institution, in which we are comparing VR-based clip placement to real-life clip placement. Apart from the advantages for the surgical team, VR may improve the informed consent process by enhancing patients' understanding of their condition and treatment options. We are currently running a randomized trial addressing this, in which we use VR models for informed patient consent (41).

## Limitations

Despite this being the largest cohort study describing VR-based surgical planning for aneurysm surgery, this study has several limitations.

First, as a retrospective analysis, it is vulnerable to selection bias and lacks randomization. Given that VR was implemented regularly from 2018 onwards, a certain selection and temporal bias were automatically introduced, as perioperative management and surgical technologies improved over time. Second, the lack of systematic access to intraoperative data limited our ability to assess specific procedural metrics such as clip placement time or the number of clip attempts. Third, while VR was introduced in a standardized fashion at our institution, keyhole approaches gained more widespread popularity, potentially confounding the observed reduction in craniotomy size. However, in our experience, VR remains an important adjunct for surgical planning, as we have completely shifted to keyhole approaches since its introduction. Fourth, more than one surgeon performed aneurysm clipping, which could introduce bias. However, the surgeons were either fellowship-trained to ensure benchmark quality or were supervised by a fellowship-trained cerebrovascular surgeon (mainly the senior author, RG) Finally, due to the retrospective design, we did not collect additional cosmetic or patient-reported quality-of-life outcomes; however, we are planning a prospective study using patient-reported outcomes to investigate this important topic.

## Conclusion

In our study, VR-based surgical planning was associated with significantly smaller craniotomy sizes and shorter hospital stays. These findings support the use of VR as a valuable adjunct in preoperative planning, potentially enabling less invasive procedures and improving intraoperative orientation. Virtual reality platforms enable a realistic, patient-specific 3D reconstruction of the neurovascular anatomy, resulting in an intraoperative déjà-vu effect for the surgeon and facilitating clip placement. However, further prospective studies are necessary to validate these benefits and assess their impact on surgical outcomes.

## Previous presentations

Parts of this study were presented at the European Association of Neurosurgical Societies Conference in Dublin in 2019, at the EANS Vascular Section Meeting in 2021 and 2022, and at the Annual Meeting of the Swiss Neurosurgical Society in 2021.

## Data Availability

The original contributions presented in the study are included in the article/supplementary material, further inquiries can be directed to the corresponding author/s.

## Supplementary material

Supplementary Video 1 Surgical planning using a virtual reality platform (SpectoMedical®) of elective aneurysm surgery clipping with patient-specific 3D models. Patient positioning, approach, and aneurysm morphology with spatial anatomy of branching vessels can be studied in detail to prepare for optimal dissection and clip placement during surgery. The video shows two example cases of an MCA and an ACOM aneurysm.
