# Supplementary material for: Minimally invasive approaches using virtual reality planning in elective aneurysm surgery
Source: Front Surg. 2025 Dec 4;12:1713243. doi: 10.3389/fsurg.2025.1713243 (PMC12711783; doi:10.3389/fsurg.2025.1713243)
Supplement: Supplementary file 1 [file Supplementaryfile1.docx]

**Supplemental Tables**

**Supplemental Table 1**: Permanent neurologic deficits due to complications

| **Permanent Complications** | |
| --- | --- |
|  | n = 6 |
| Anosmia after Acom aneurysm clipping | 1 (0.6) |
| posterior ischemic optic neuropathy after paraophthalmic ICA aneurysm clipping | 1 (0.6) |
| Basal ganglia infarction with permanent hemisyndrome after MCA aneurysm clipping | 2 (1.2) |
| ICH with hemisyndrome after MCA aneurysm clipping | 1 (0.6) |
| Intraoperative aneurysm rupture requiring decompressive craniectomy during AchoA aneurysm clipping | 1 (0.6) |

ICH = intracerebral hematoma

**Supplemental Figures**

**Supp Figure 1**: Linear regression of the significant positive association of operating time with the approach size (β = 0.86, SE = 0.43, t(df) = 1.98, p = 0.049).

**Supp Figure 2:** Aneurysm location is significantly associated with the operating time.
